# A novel rapid (20-minute) IL-6 release assay using blood mononuclear cells of patients with various clinical forms of drug induced skin injuries

**DOI:** 10.1186/1939-4551-8-1

**Published:** 2015-01-09

**Authors:** Joseph M Baló-Banga, Katalin Schweitzer, Susan Lakatos, Sándor Sipka

**Affiliations:** Department of Dermatology, Medical Center of Hungarian Defense Forces, Podmaniczky u. 109-111, Budapest, H-1062 Hungary; Department of Pathophysiology, Medical Center of Hungarian Defense Forces, Róbert Károly krt. 44, Budapest, H-1134 Hungary; Division of Clinical Immunology, University of Debrecen, Móricz Zs. u. 22, Debrecen, 4032 Hungary

**Keywords:** IL-6, TNF-alpha, T-lymphocytes, Drug-induced skin injury, Adverse drug reactions, Preformed cytokines’ release

## Abstract

**Background:**

IL-6 is a pro-inflammatory cytokine which has many well-defined effects. Its synthesis and release from mononuclear cells of drug-sensitized patients was related before to *in vitro* drug-allergy diagnostics but has not yet been studied in detail.

**Methods:**

The specific release of preformed IL-6 from peripheral blood mononuclear cells (PBMC) after 20 minutes incubation with 0.15–0.5 μM of pure drugs was measured in two groups of drug-allergy suspected donors (159) and respective controls (48). IL-6, TNF-alpha, IL-2, IL-4, IFN-gamma have been measured from cell supernatants by ELISA or by cytometric bead assay. Epicutaneous, intradermal and systemic provocation tests were performed to prove or disprove culprit substances (203 *in vivo* against 482 *in vitro* tests). *T*-test (paired and unpaired); chi2 contingency table; Z statistics and McNemar’s test were used to evaluate results.

**Results:**

Concanavalin A as positive control released IL-6 from PBMC in linear concentration and exponential time dependent fashion (up to 60 minutes) pointing to the existence of a preformed pool of this cytokine.

Preformed IL-6 released at any of 4 standard drug dilutions tested, above 50% over their diluents’ levels significantly correlated with the patients’ history on drug-induced hypersensitivity symptoms and with *in vivo* tests.

Sensitivity of 85.4% and specificity of 82.4% of the IL-6 release assay were found. The 20′ drop in release of TNF-alpha had no diagnostic importance; it has accompanied increased IL-6 release. IL-2, IL-4 and IFN-gamma were undetectable in 20 minutes supernatants. IL-6 release depended on the clinical phenotype but not on the eliciting drug(s) in the molecular mass range of 76–4000 Da. Reactivity of mononuclear cells at the lowest or at multiple drug test concentrations reflected clinical severity per diagnoses and according to area of skin involvement.

**Conclusion:**

This rapid test is applicable to detect a wide scale of drug hypersensitivity.

## Background

It is generally accepted that about 20% of all adverse drug reactions (ADR) are immunologically mediated [1, 2]. The majority of these reactions has skin manifestations [3]. The diversity of humoral and cellular mechanisms motivated Pichler to study the different T-cell subsets at certain well-defined clinical pictures. In addition to hapten and prohapten presentation of small molecular drugs the concept of pharmacological interaction (p-i) has emerged [4]. This concept explains for the rapid elicitation of generalized symptoms due to the binding of unchanged drugs to TCR and MHC resulting in activation of mediators and cytokines. Studies were designed to identify and exploit the measurement of those cytokines in search for culprit drugs. These tests have measured *de novo* synthesized molecules from cultivated cells’ supernatants [2, 5, 6].

IL-6 a 22–27 kDa peptide is involved in numerous cellular and molecular mechanisms of inflammation including T and B cell activation and synthesis of acute phase proteins by hepatocytes. IL-6 binding to its membrane-bound receptor (IL-6R) results in signal transduction [7]. IL-6 type cytokines bind to membrane receptors activating both the JAK/STAT and the MAPK cascades [8].

In earlier studies we and others found that short term *ex vivo* incubation of PBMCs with standard dilutions of sensitizing drugs has changed the chromatin structure of lymphocytes in a specific manner [1, 9]. After a 20-minute incubation with the offending drug release of IL-1α, IL-1β, IL-6 cytokines could be measured concomitant with the structure change of chromatin. Chromatin "relaxation" measured by morphometry correlated best with the prompt release of IL-6 [9]. Our preliminary results on 45 ADR suspect patients with five controls were promising [10]. Lochmatter et al. [2] cultivated PBMCs of control donors and of patients with well defined drug allergies for 24–72 hours with aminopenicillins or sulphonamides according to their histories. These PBMCs have shown significant IL-6 release only in AMX sensitive patients. Sixteen other cytokines/chemokines were tested simultaneously as well, out of them IL-5, IFN-γ, IL-13 and IL-2 seemed to be suitable only in combination for diagnostic purposes.

The aim of the present study was to limit the plethora of measurements to a single cytokine, namely to IL-6 and standardize the sensitivity and specificity of the procedure. Of note, preformed cytokines were not known to operate in mononuclear cells contrary to eosinophils [11] and mast cells [12]. Furthermore, we aimed to demonstrate that early IL-6 release is specific of the drug causing immune-mediated reactions, and it does not depend on the type or structure of drug or on the phenotype of the skin allergic reactions.

## Methods

### Patients and controls

Patients were seen and treated by our group at the Department of Dermatology or as out-patients in the ADR Clinic of the Military Hospital in Budapest. Ninety eight patients with suspected drug hypersensitivity were studied between 2007 and 2011. Both immediate and delayed type allergies were represented (Table 1). There were 80 women and 18 men, their mean age was 49.9 ± 18.9 (SD). The patients fell into definitive (46%), probable (20%), possible (21%), not related (11%), and impossible (2%) categories as defined by Karch and Lasagna [13]. In 24 control subjects the drugs as offending substances could be ruled out (categories impossible or not related = 98%). These groups together were marked as "*Test A*". Tests were carried out in a currently symptom-free state as usual between 4 weeks and 1 year after cessation of therapy. Between 2005 and 2007 sixty-one patients and 24 control subjects were seen and tested under identical conditions and selection criteria. In this group there were 49 women and 12 men, mean age was 52.4 ± 17.9 (SD). According their history 45% were definitively, 19% probably, 19% possibly allergic and 17% fell into the not related category. None were marked as "impossible". Their matching control group comprised 20 women and 4 men Out of 22 (92%) 14 persons belonged to the impossible and 8 persons to not related symptoms revealed while 2 persons were possibly allergic. These groups were designated as "*Test B*". Assay conditions were different for the two groups. All gave their informed consent and the study was approved by the Ethical Committee of the Medical Center of the Hungarian Defense Forces.

**Table 1:** **Distribution of clinical manifestations of drug hypersensitivity from "** ***Test A*** **" group (values are given in % of cases)**

		IL-6 release pattern			
Phenotypes		with peak at 0.15 μM, (n = 37 tests) I	with peak at 0.35 μM, (n = 67 tests) II	with peak at 0.5 μM, (n = 38 tests) III	with 2 or more positivities (n = 65 tests) IV
1	Generalized urticaria ± ANO^2^	17	18	11	18
2	Systemic Anaphylaxis ± ANO	23	24	24	28
3	DRESS^3^ (**culprit**/non-culprit^*^)	**3**	0	3^*^	0
4	Generalized MPE^4^ (>18%)	14	16	8	20
5	Localized MPE (<18%)	6	4	8	5
6	Disseminated fixed drug eruption	1	2	0	0
7	Erythema multiforme	0	2	0	2
8	Asthma, severe itch	1	0	0	0
9	Generalized disseminated dermatitis	9	2	11	8
10	Small patchy urticaria	3	9	8	5
11	Localized ANO	14	17	18	8
12	Leg dermatits ± purpurae	0	3	3	5
13	Circumscribed vesiculae	3	0	3	0
14	Erythema annulare centrifugum/E.nodosum	6	3	3	1

^2^ANO- angioneurotic edema.

^3^DRESS –drug rash with eosinophilia and systemic symptoms.

^4^MPE- maculopapular exanthema.

*non-culprit drug representation of a single case.

Boldface number in column II marks culprit.

### *In vitro*tests

#### Drugs and mitogens

Non-toxic (final) drug concentrations were used in each test series 0.15; 0.25; 0.35 and 0.50 μM, prepared freshly from pure substances or diluted from sterile injections or other suitable liquid drug formulations. The molecular masses of drugs investigated varied between 76 (Propylene glycol) and ~4000 Da (Enoxaparin sodium). The pure drugs selected according to the patients’ history were either gifts of certain pharmaceutical companies or had been purchased from LGC Standards GmbH (Wesel, Germany). To obtain *in vitro* positive controls the cells were stimulated either with PHA-P (PHA_1_ 168 μg/ml; PHA_2_ 335 μg/ml, Sigma-Aldrich Co.) or with Con A (Sigma-Aldrich, type 6) tested at 5 to 300 μg/ml concentrations.

#### Separation of PBMC

Was done by using Ficoll-Paque™ (Amersham, Biosciences) as described [14] and washed twice with PBS containing 2 mM of EDTA and 0.5% w/v of BSA. The cells were then re-suspended in modified Dulbecco’s MEM [15] containing 100 mM NaCl, 24 mM KCl, 10–10 mM CaCl2 and MgCl2, and 11 mM glucose, pH: 7.2 (*Test A incubation medium*). In earlier experiments a different MEM solution was used containing 145 mM NaCl, 21 mM KCl, and 0.7- 0.7 mM CaCl2 and MgCl2 and 11 mM glucose, pH: 7.2 (*Test B incubation medium*). The incubation of 1.1 × 10^6^/ml cells without any plasma or serum was carried out in 450 μl aliquots for 20 min at 37°C with drugs or mitogens dissolved in 50 μl of solvent. The incubation was terminated by placing the tubes into crushed ice and then the fluid was centrifuged at 30–50 × g for 6 min. The water-clear supernatants were carefully removed and kept frozen at -80°C until cytokine determinations.

#### Detection of IL-6 in the cell-free supernatants

IL-6 was determined in the cell-free supernatants by solid phase immunoassay (Diagnosticum Ltd., Hungary) according to the manufacturer’s instructions, as described earlier [16]. In addition, both incubations with polyclonal- and monoclonal anti-IL-6 antibodies were performed under mild shaking at 37°C for 60 min. The calibration curve was linear between 10 and 700 pg/ml IL-6 concentrations (0.951 < R2 < 0.988). O.D. values falling below or above this range were extrapolated.

*Cytotoxicity measurements* were performed on selected cell-free supernatants using the automated (Roche Modular T-800) determination of LDH.

*Simultaneous Detection* of IL-2, IL-4, IL-6, IL-10, TNF-α and IFN-γ was performed with the BD-CBA Human Th1/Th2 Cytokine Kit II according to the manufacturer’s instruction (Becton Dickinson, Franklin Lakes, NJ, USA). Briefly, 50 μl of mixed human Th1/Th2 cytokine capture beads and 50 μl of phycoerythrin labeled detection reagent were incubated either with 50 μl of each test sample or with 50 μl of the human Th1/Th2 cytokine standard dilutions for 3 hours at room temperature in dark. After a brief washing (200 × g, 5 min), samples were run on a BD- FACS Array flow cytometer. Data acquisition and analysis were performed with the BD™ CBA software.

### *In vivo*tests

Drug patch tests were done either with 10% w/w pure substances, or less frequently, with 30% w/w ground powder of tablets in petrolatum. Curatest™ (Brial GmbH, Germany) adhesive chambers were used. Occasionally 5–10% w/v solutions in distilled water were prepared. Results were read after 20 min, 48 hrs, 72–96 hrs. Intradermal tests were prepared under sterile conditions. Pure drug substances or injection formulations (eye drops) were diluted in 2 steps to obtain 1 × 10^-3^ M solutions in PBS. Water insoluble substances were first dissolved in DMSO and diluted further with PBS to obtain the desired concentrations. The concentration of DMSO never exceeded 1% v/v. Negative (diluent) and positive controls (Histamine 0.1 mg/ml) were included with all tests. Injections (0.04 ml) were placed in the volar skin of forearm. Results were recorded at 20 min, 90 min and 24 hrs. Positivity was only accepted if 10^-3^ M concentration gave >3 mm papules/wheals increasing in time with or without a red halo. Any skin reactions obtained only at higher than 10^-3^ M of drugs or additive substances were considered as "irritative".

Drug provocation tests were performed under conditions set by the ENDA and by the EAACI group on drug hypersensitivity [17]. Incremental doses were given orally [1, 17] or subcutaneously under strict control (with emergency room coverage) over 3 hrs. in the ward, followed by a 24 hour phone contact. The tests were performed parallel to *in vitro* results even after severe reactions or in doubtful cases to differentiate between hypersensitivity and e.g. vagal reaction due to local anaesthetics. Positivity was accepted if skin or systemic symptoms arose (mainly within the close observation period).

### Data analysis

Statistical significance was determined by the *t*-test for both paired and unpaired data. For analysis of the morbidity rates the χ2 and Z-statistics were employed. A p < 0.05 value was considered to be statistically significant. Determination of diagnostic efficacy including specificity and sensitivity related to *in vivo* exposures were generated by McNemar’s test.

## Results

The numbers of complete tests in the two groups (*with Test A and Test B solutions*) are shown in Table 2. The total number of all *in vitro* diagnostic test series based on IL-6 release was 482. A test was considered positive if the concentration of IL-6 in the supernatant of PBMCs incubated with the drug was higher by 50% than in its control counterpart at any concentration. Cases where increase of IL-6 was exactly 50% at any concentration were considered as doubtful.

**Table 2:** **Summary of the tested groups**

Groups	" ***Test A*** " solution	" ***Test B*** " solution
Number (N) of controls	24	24
N of tests in controls	50	49
N of negative tests in controls	48	48
Doubtful and positive tests in control group	2	1
N of suspect patients	98	61
N of tests in group of suspect patients	266	121
Positive tests in group of suspect patients	151	32
Negative tests in group of suspect patients	113	87
Doubtful tests in group of suspect donors	2	2
(Test/person) for control group	2.1	2.1
(Test/person) for suspect patients	2.7	1.9
Total N of tests	316	166

"*Test A*": 100 mM NaCl, 24 mM KCl, 10 mM CaCl_2_, 10 mM MgCl_2_, 11 mM glucose; pH:7.2.

"*Test B*": 145 mM NaCl, 21 mM KCl, 0.7 mM CaCl_2_, 0.7 mM MgCl_2_, 11 mM glucose; pH:7.2.

### IL-6 release caused by positive controls (Figure 1)

**Figure 1:**
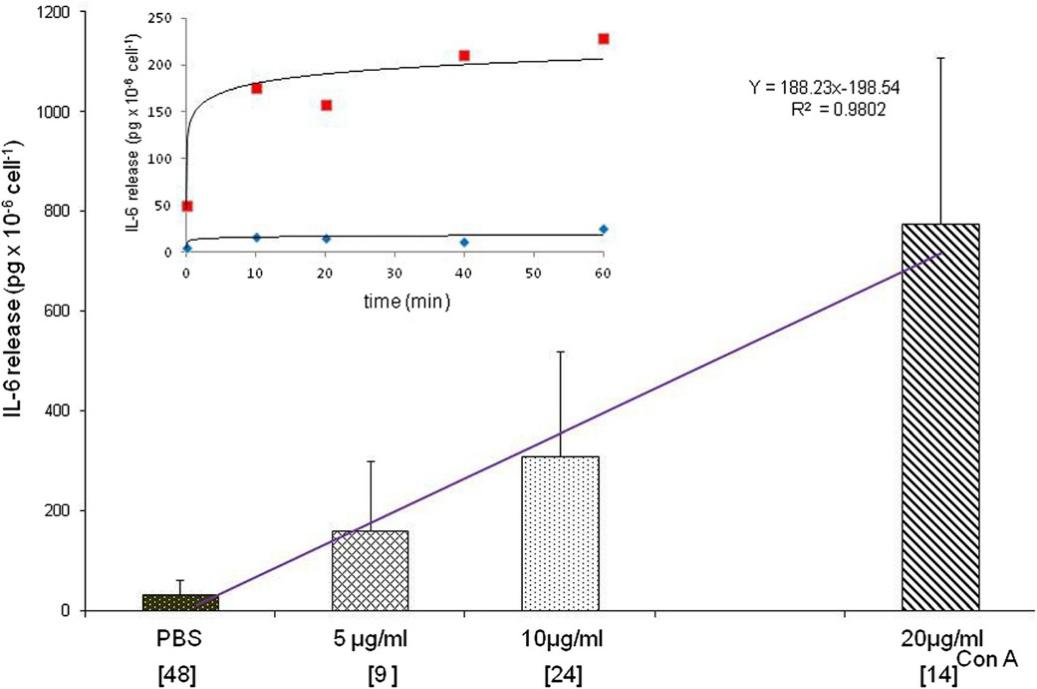
**Effect of ConA on the release of IL-6 from mononuclear cells of patients after 20-min incubation with "** ***Test A*** **"** ***solution*** **.** The columns represent mean ± SD. [Brackets under abscissa indicate numbers of tests at different concentrations]. The value of 2197 ± 268 pg × 10^-6^ cells^-1^ was obtained at 300 μg/ml ConA. Insert: fitted time course of the mean values from 2 independent experiments using 5 μg/ml ConA on 2 non-allergic persons’ cells (red: ConA, blue: PBS).

The dose response of mononuclear cells to the mitogen Con A was linear between 0–20 μg/ml. The time course experiments have shown that the IL-6 release is almost complete by the 20^th^ min of Con A stimulation. Five μg/ml was used as positive control. However, much higher doses of PHA-P were needed. Two concentrations, 168 μg/ml (PHA_1_) and 337 μg/ml (PHA_2_) were tested and PHA_1_ was used. In time course experiments a plateau was reached between 10 and 50 minutes of incubation which declined thereafter (data not shown in details).

### Time dependence of the drug specific IL-6 release

**Figure 2:**
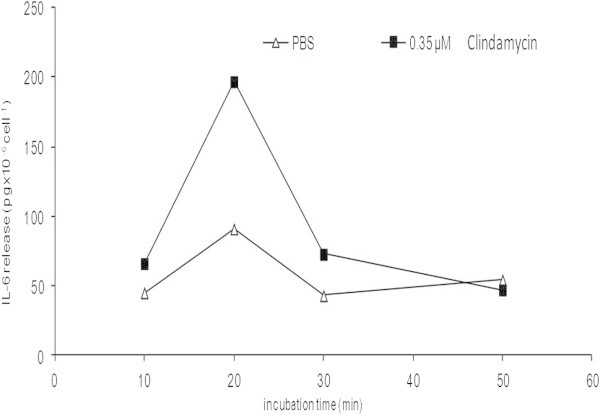
**Time course of drug-induced IL-6 release from PBMCs of an allergic patient (clindamycin, phenotype ANO) 6 months after the event.** The drug was taken orally as monotherapy. Localized edematous rash has developed around the wrist and on dorsa of hands 30 min. after repeated intake. Results of 2 independent experiments with 6-week interval yielded 196 and 198 pg × 10^-6^ cells at 0.35 μM (the points coincide).

Typical time dependence of IL-6 release upon drug challenge of PBMCs of a hypersensitive person is shown in Figure 2, demonstrating that the 20-minute incubation time resulted in maximal release.

### IL-6 release from PBMCs incubated for 20 minutes with different drug concentrations

**Figure 3:**
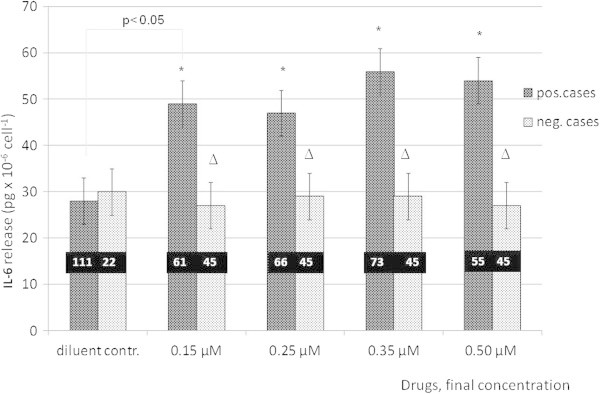
**IL-6 release from PBMCs upon different drug concentrations of various drugs in positively and negatively reacting groups of the cohort incubated with "** ***Test A*** **" medium.** Positivity: >50% increase in IL-6 release at any drug concentration relative diluent control. Stars indicate statistically significant differences) between negative control samples and positively tested cases, (p < 0.05) and between negative and positive cases (p < 0.005).

The average IL-6 release increased significantly over the background level at all tested drug concentrations only in the positive test series. The mean increases of IL-6 release were 75% at 0.15 μM, 69% at 0.25 μM, 103% at 0.35 μM and 96% at 0.5 μM final concentrations of various drugs (Figure 3). Both the highest mean cytokine release and the highest number of positive results were found at 0.35 μM drug concentration in *Test A medium*. In *Test B medium* the highest IL-6 release and the highest number of positive results were detected at 0.15 μM and at 0.25 μM drug concentrations, respectively. In negative test series and in controls the average IL-6 release was not significantly different from that of the diluents at any drug concentrations tested. LDH concentrations were low (1–3 U/l) both for the positive and negative cases proving the presence of intact cells. Those samples containing damaged cells upon separation (LDH concentration 130–150 U/l) were excluded from further evaluation.

### Reliability of IL-6 release measurements in "*Test A*" and "*Test B*" groups

Table 3 summarizes the results at 10 controls and 50 patients of Test A compared to 12 controls and 36 patients of *Test B* groups tested *in vitro* -*in vivo* simultaneously. The parallel tests varied between 1 and 5 per individual persons. Some patients were tested *in vivo* by different drugs or with the same drug using different tests. In group A there were 99 tests out of which 13 in controls, and 70 in the patients gave identical results. Among concordant positive tests there were 63% mild systemic reactions in 25 instances due to oral provocation and in one case as the complication of positive patch testing. In 2 patients anaphylaxis (grade II) occurred upon intravenous administration of ferric sodium gluconate. IL-6 release was later positive to 3 differently colored monocomponent ferric oxides yielding thus 2 × 3 matching results. In this group 20% identical patch tests and 17% intradermal tests were obtained. Among matching negative tests 69% provocation 9,5% patch and 21,5% intradermal were noted. Within "*Test B*" group there were 85 tests out of which 19 in the control group and 49 in the patient group gave identical results. One *in vitro* test was false positive but this person used inhalant steroid for asthma while tolerating ropivacain the substance, tested.

**Table 3:** **Evaluation of the parallel** ***in vitro*** **-** ***in vivo*** **tests in the two groups**

Parameters	" ***Test A*** " solution	%	" ***Test B*** " solution	%
Total N of parallel tests	99		85	
Both negative	42		54	
Both positive	41		12	
Neg. IL-6, pos. in vivo*	7		15	
Pos. IL-6, neg. in vivo**	9		5	
Sensitivity		85.4		44.4
Specificity		82.4		93.1
Reliability		83.8		77.6
Positive predictive value		82.0		70.6
Negative predictive value		85.7		78.2

^*^false negative.

^**^false positive.

"*Test A*": 100 mM NaCl, 24 mM KCl, 10 mM CaCl_2_, 10 mM MgCl_2_, 11 mM glucose; pH:7.2.

"*Test B*": 145 mM NaCl, 21 mM KCl, 0.7 mM CaCl_2_, 0.7 mM MgCl_2_, 11 mM glucose; pH:7.2.

*in vivo: "*Test A*": oral provocation 2, intradermal 5 tests were positive.

*in vivo: "*Test B*": oral provocation 5, epicutaneous 2, intradermal 8 tests were positive.

**in vivo: "*Test A*": oral provocation 5, epicutaneous 1, intradermal 3 tests were negative.

**in vivo: "*Test B*": oral provocation 3, intradermal 2 tests were negative.

Among concordant positive tests 43% were oral, sc or iv provocations, 14% patches and 43% intradermal ones. Out of the matching negative results 49% were due to provocation, 13% due to patch testing and 37% intradermal testing. The non-matching tests are marked with asterisks in Table 3. The *in vitro* test sensitivity in group "*Test A*" was markedly higher than in "*Test B*" (85.4% versus 44.4%). In contrary, the test specificity was higher in the "*Test B*" group (93.1%) than in the "*Test A*" (82. 4%). However, both overall reliability and predictive values were higher in the "*Test A*" than in "*Test B*" group.

### Distribution of the pharmacological classes of the tested drugs in the patients and in the control groups

**Figure 4:**
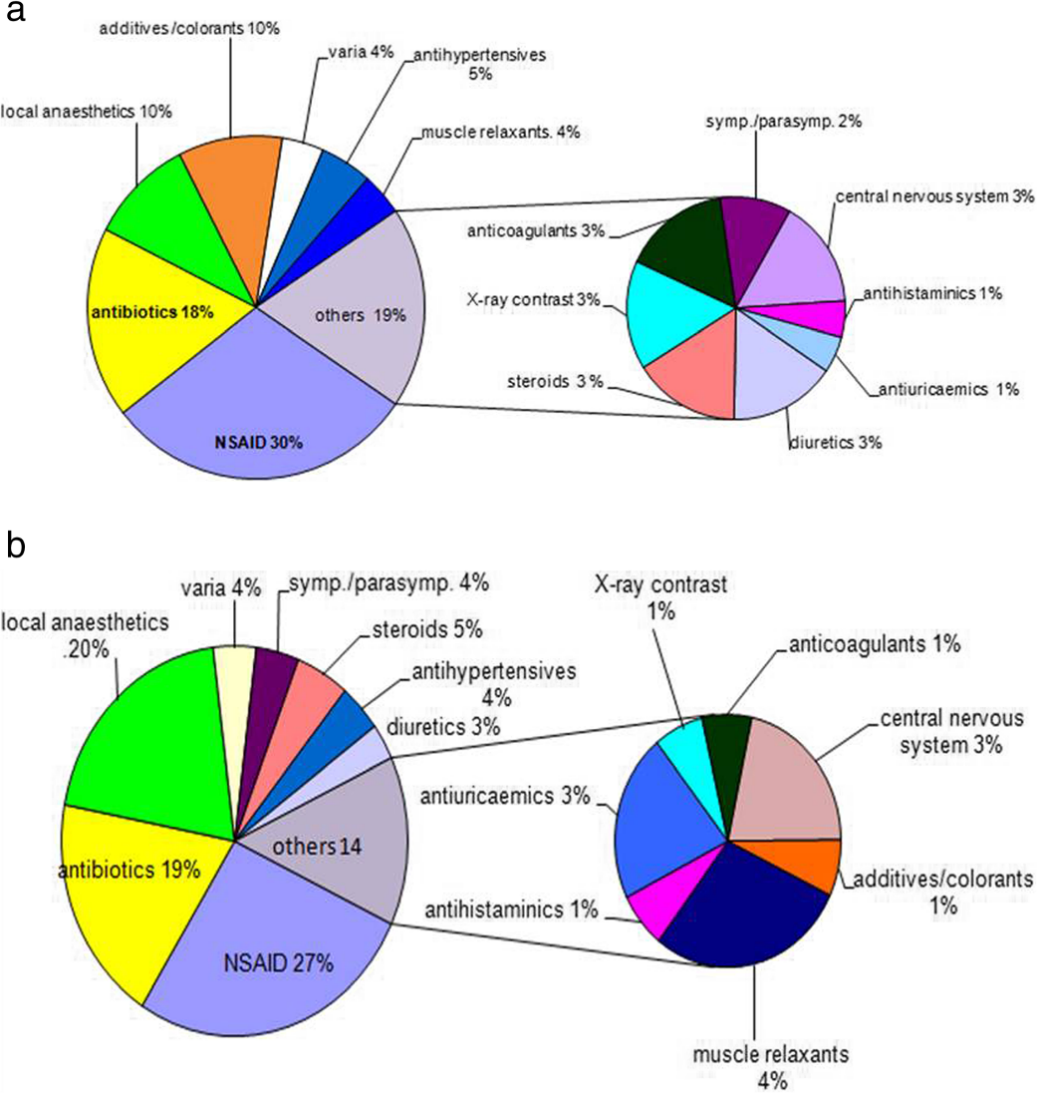
**Distribution of drugs among different pharmacological classes.** **a**: eliciting positive IL-6 release test results (n = 43). **b**: tested within the control group (n=40). The numbers of individual drugs tested are higher (~70). Glibenclamide peripheral vasodilators and negative tests with acetylcystein are listed among "varia".

The two dominant classes were antibiotics and non-steroidal anti-inflammatory drugs (Figure 4a-b) in both test series. According to the individual histories 16 drug classes were tested both in the ADR-suspect groups and in the matching controls. Among the additives, iron oxides (E172) used to stain tablets were most often tested, and both positive and negative results were obtained. Sixteen additional drugs, among them enalapril (ANO and cough in history) gave only negative results. Some biologicals and cytostatic agents could not be evaluated although their molecular mass fell within the test range. In addition to drugs purified endotoxin (lipopolysaccharide) was tested in two independent experiments using serial dilutions. No additional IL-6 release exceeding PBS controls was detected.

### Clinical diagnoses and positive IL-6 release at different standard drug concentrations

The results for *Test A medium* are listed in Table 1. The relative frequency of single IL-6 positivity within the test series is shown in columns I, II, III, while those with multiple positive IL-6 release appear in column IV. Comparing data of Table 1 with those in Figure 3, positive results comprised 61% (37/61) at 0.15 μM, 38% (25/66) at 0.25 μM, 62% (45/73) at 0.35 μM and 69% (38/55) at 0.5 μM drug concentrations, respectively. The number of tests with more than one drug concentration causing positive IL-6 release was 65 out of a total of 153 positive tests (42,5%). These results reflected the more widespread and severe skin and mucosal lesions of ADR (except for DRESS in one case). Using "Z" test, combined data of lines 1,2,4,6,7,9 (column IV Table 1) were compared with those of lines 5,10–14 respectively, of column III representing less severe localized forms of ADR. The binomial distributions were significantly different (p < 0.001) unlike in columns I and III where no significant differences were found. Multiple IL-6 releases differentiated light from severe or widespread manifestations. These skin injuries (lines 1–4, 6–9; including a case of DRESS but only with culprit drug) and light and circumscribed ones differed significantly ("Z" test, p < 0.05) in terms of drug concentrations eliciting maximal IL-6 release (column I). These severe generalized manifestations caused mostly positive IL-6 release at the lowest drug concentration tested. Identical lines (5, 10–14) of columns I, III against IV were compared by χ^2^ test. No significant differences were found. Single peak positivities at 0.35 μM (column II) showed a "mixed" pattern; both widespread severe and localized milder forms were represented here.

### Simultaneous release of IL-6, TNF-α (Figure 5) and IL-10

**Figure 5:**
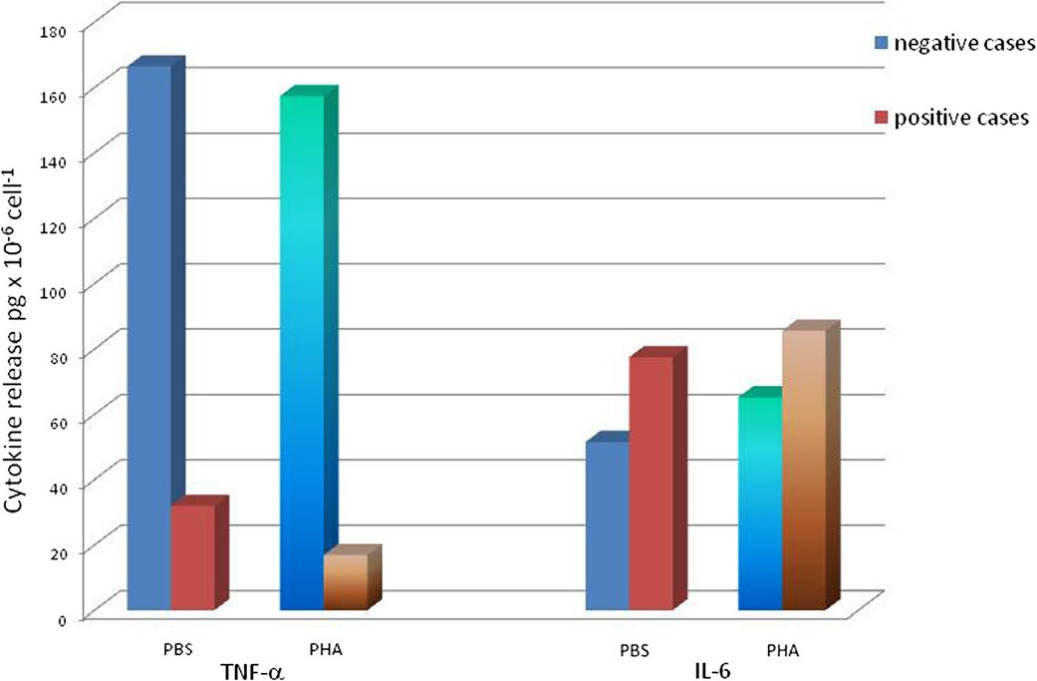
**Mean cytokine releases stimulated by PHA-P (168 μg/ml) after 20-min incubation with "** ***Test B solution*** **" compared to controls.** Non-allergic test series (n = 6) appear for both cytokines, TNFα and IL-6 in blue, allergic test series (n = 4) in light brown color.

**Figure 6:**
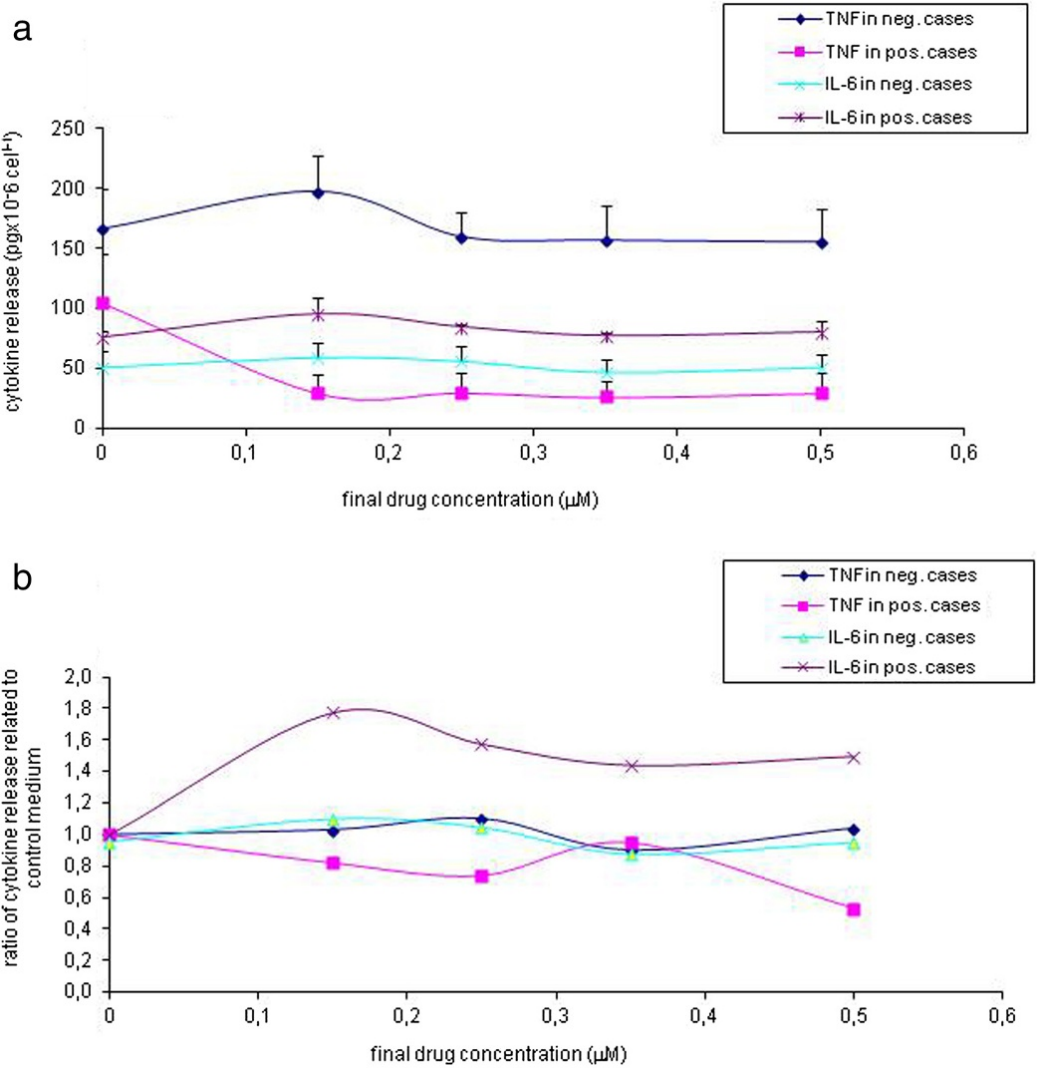
**TNFα and IL-6 release from PBMCs incubated with "** ***Test B solutions*** **" elicited by different drug concentrations measured by the CBA Th1-Th2 cytokine kit in a total of 6 negative and 4 positive assays.** **a**: cytokine concentrations (mean +- S.E.M.); **b**: relative cytokine release normalized individually by their corresponding control values.

Four patients with altogether 8 drugs and two control donors with exclusion of all types of ADR and negative oral provocations were tested. Concentrations of TNF-α and IL-6 were simultaneously determined by the human Th_1_/Th_2_ cytokine kit, together with IL-2, IL-4_-_ and IFN-γ from the 20-minute supernatants of the PBMCs incubated with drugs or medium (*Test B*). There have been no measurable amounts of IL-2, IL-4 and IFN-γ in any of the 10 test series. TNF-α and IL-6 were present though. In six tests with negative IL-6 results PHA stimulation resulted in lowered TNF-α and increased IL-6 releases (Figure 5). Both control cases and tests of patients with nonreactive drugs as judged by their low IL-6 release exhibited high TNF-α output. In cases where IL-6 release test was positive the TNF-α release was significantly lower than in negative cases at all drug concentrations. In positive cases the highest IL-6 release was at 0.15 μM drug concentration (Figure 6a). This opposite behavior in the release of the two inflammatory cytokines can even better visualized in relation of their own background (diluents) values (Figure 6b).

IL-10 and IL-6 were simultaneously measured in 13 tests of 6 donors in the 20-minute supernatants. Although upon challenge with PHA_1_ all donors’ PBMCs released both IL-10 (80 pg/ml in average) and IL-6 (110 pg/ml in average) no detectable amount of IL-10 was released either in cases of positive (4 different drugs) or negative IL-6 responses (not shown in details).

## Discussion

To obtain positive controls for the IL-6 release from PBMCs we used PHA exceeding about 20 times the amounts of those claimed to stimulate lymphocyte proliferation in 3–5-day cultures in the presence of serum [18]. On the other hand ConA was active in the same range as in LTT and increased the release of IL-6 in a dose- and time dependent manner (Figure 1). However, considerable inter- and intraindividual variations were experienced. Con A (5 μg/ml) seemed to act as proper positive control. PHA in 168 μg/ml concentration resulting in only 1–5% release of the expected preformed IL-6 from PBMC suspensions has acted in a similar limited fashion as the culprit drugs within the selected range. The question arises whether a small fraction of cells could account for the 1–5% of IL-6 release from a much larger (>2000 pg × 10^-6^cell^-1^) intracellular pool in T- lymphocytes as concluded from the extended dose response results obtained with Con A (2197 ± 268 SD at 300 μg/ml upon 6 experiments) or had the release occurred uniformly.

The time course of IL-6 release upon drug challenge of PBMCs suggests that IL-6 has originated from a preformed pool. The timing of the earliest onset of IL-6 synthesis was addressed by McHugh et al. [19]. They demonstrated that PHA has initiated *de novo* IL-6 production in PBMCs both of atopic and control donors after 4 hours. The maximal amount approximated 22–36 × 10^3^ pg/ml. Thus indirect evidence suggests a preformed pool size of one tenth of this magnitude.

The search for a more rapid and less cumbersome test replacing LTT in the diagnosis of a wide range of drug hypersensitivities has resulted in the detection of CD69 up regulation on a small group of CD4+ T-cells after 48 hrs of incubation [20]. The results are in good agreement with our findings.

IL-6 secretion has dropped in damaged cell suspensions (LDH increased in the supernatants) regardless of mitogen or to any drug concentration. Under "usual" assay conditions LDH was at detection limit. Thus, cytokine release due to cellular damage or to direct drug toxicity appears unlikely. Recent results on mouse mast cells have proven that specific desensitization of the animals either to ovalbumin or dinitrophenol blocked both the TNF-α and IL-6 releases from cells upon 30-minute and 4-hour *in vitro* challenges [12].

Comparing the *in vitro* vs. *in vivo* data for groups tested by *Test A* or *Test B* incubation media (Table 3) the importance of the signaling process became evident. In the early phase of these studies Dulbecco’s rather simple solution enriched with 11 mM glucose and supplemented with low concentrations of divalent cations (0.7 mM Ca2+ and Mg2+) was used in order to avoid cell-clumping [15]. The low test sensitivity shed light on the importance optimizing assay conditions. Raising the concentrations of Ca^2+^ and Mg^2+^ by fifteen-fold within the test medium resulted in a shift of the maximal IL-6 release from 0.15 and 0.25 to 0.35 and 0.5 μM (Figure 3). In *Test B* the increase of IL-6 release was only 5% compared to 103% and 96% obtained in *Test A medium at* 0.35 and 0.5 μM, respectively. The concentrations accounting for test positivity were as follows: 0.15 μM, 9 cases, 0.25 μM, 6 cases, 0.35 μM, 5 cases and 0.5 μM, 7 cases. Using *Test B* only 5 out of 122 tests had multiple positive readings against 65 out of 151 obtained with *Test A* (Table 1). The lack of IL-6 release at six out of the overall 13 false negativities could be attributed to the low Ca^2+^ and Mg^2+^ at 0.35 and 0.5 μM drug concentrations. This means that the test sensitivity (against *in vivo* results) depends on the proper divalent cationic concentrations.

In our diagnostic groups there were both non-widespread and not life-threatening eruptions together with some serious and potentially lethal reactions (anaphylaxis grade II-III, DRESS). Beyond drugs most of them could have been caused by other elicitors too e.g. by infections [3, 21, 22]. Generalized disseminated dermatitis was clinically different from MPE. Stasis dermatitis of the legs is often aggravated by sensitization to drugs. Both toxic epidermal necrolysis, or acute generalized erythematous pustulosis (AGEP) have been tested earlier, but not with the standard media "A" or "B". Thus, results were not included in Table 1. Multiple positive results with drugs suspected have been obtained though.

The concept of using multiple drug concentrations instead of only one was crucial to establish significant positive correlation between the severity (although not scored) and skin area involvement in most drug hypersensitivity related clinical phenotypes (Table 1), which had previously not been proven by any tests [5, 6, 22], but were suggested by the 20-minute chromatin activation results [1, 9]. Using molar concentrations, enables one to compare clinical manifestations elicited by chemically different drugs (between 76 and 4000 DA) since the number of tested molecules reacting with cellular receptors are identical. The receptor equivalence is also concordant with the p-i concept of Pichler [4]. The rationale for selecting the lowest and the highest concentrations from the dilution series was to prove the inverse correlation between the drug concentration resulting in maximal IL-6 release and the severity of clinical reaction but only with the culprit drug. For the generalized widespread lesions the single peak positivity frequencies at 0.15 μM are close to those of multiple positivity by comparing column I with column IV in Table 1. Our DRESS syndrome case e.g. with multivalent drug hypersensitivity had highly elevated IL-6 release with the relevant culprit drug at 0.15 μM, whereas another non-culprit drug (by history) has caused the peak exclusively at 0.5 μM. The *in vivo* tests are known to have different sensitivities and only provocations are considered as gold standards. Their percentages to evaluate any *in vitro* tests are important. The ratios of provocations were higher in *Test A* group than in *Test B*. Their use was not restricted only to prove negativity of *in vitro* tests. In many patients *in vivo* tests were performed successively starting with patch tests followed by intradermals which we attempted to standardize as well [23]. Our data have revealed that provocations against intradermal tests with 10^-3^ M drug solutions had 33% less positive results.

Recent results obtained on abacavir reactive CD8+ T-cell clones isolated from genetically susceptive HLA-B*5701+ individuals showed that their TCR exerted different avidity. Some of them reacted instantly to the drug in solution [24].

Our tests with methothrexate were highly positive at all concentrations in 3 treated rheumatological patients after widespread rashes. The same test resulted in false positivity in the two controls (who never took this antimetabolite before) at least in one drug concentration. LTT results were not satisfactory with this drug, either [25]. We recommend to perform IL-6 release assays with cytostatics emerging from the patients’ history but keeping in mind that no published data are available yet. A possible candidate could be azathioprin [26]. For some biologicals (heparin and derivates) the test was proven of value [27] but the lack of experience with receptor antagonists, cytokine therapies and especially with high molecular weight proteins should be emphasized. The negativity of enalapril in 2 suspect cases reacting with cough and swelling points to the fact that in subjects with idiosyncrasy to ACE inhibitors none of the usual allergic mechanisms appear to be involved, therefore these drugs should be excluded from the tests^a^.

The immunological synapse concept has emerged in recent years [28]. This might explain local signaling as early as 15 minutes after the onset of a close cell to cell contact in response to 1 μM antigenic peptide as observed by total internal reflection microscopy or suggested by our earlier studies on chromatin birefringence changes using polarized light microscopy [1, 5, 9]. The α-chain of IL-6 receptor binds both the soluble and membrane bound forms of its ligand. It is unable however, to induce signaling by itself. Trans-signaling occurs if gp 130, another membrane constituent binds to IL-6Rα. This may help to extend IL-6 stimulation to cells that lack IL-6 receptors but contain gp 130 [29]. IL-6/sIL-6R complexes regulate the inflammatory state, e.g. by inhibition of TNF- α [30]. In those early experiments in which exogeneous IL-6 was introduced to humans, induction of both IL-1Rα that bound IL-1β and circulating TNF receptors was shown [31]. These factors might switch off the early apoptosis induced by certain drug concentrations, thus possibly being responsible for tolerance as well [32]. This cytokine antagonism might be inferred on drug specific cytokine release from the results demonstrated in Figures 5 and 6 as well. From earlier experiments of PBMCs in drug hypersensitive patients. a basic release of 100–300 pgxml^-1^ TNF-α was evident at 24 hrs [2]. There are no data available for the time interval between 0–60 min. The authors have shown a time dependent decrease of TNF-α at 48 and 72 hours in unstimulated samples but inconsistent data for the culprit drugs of the sulfonamide as compared to aminopenicillin drug antigens have emerged. Similarly, the positive control (5 μgxml^-1^ tetanus toxoid), used has resulted in a tenfold drop in aminopenicillin sensitized patients’ TNF-α releases against unsignificant elevation at sulfonamide allergic ones from 24 to 72 hrs [2]. Our results point to an antagonism between the two early inflammatory cytokines. This seemed to be specific and concentration dependent with marked differences between sensitizing and tolerated drugs. Moreover, the direction of the changes in cytokine releases due to positive control polyclonal mitogen PHA and specific sensitizing drugs was the same. These results would need further corroborative studies, though.

Our data support the view that sensitivity to a given drug may well be reflected and quantified by the "early" IL-6 release from patients’ PBMCs. Thus, we suggest to measure as an appropriate rapid *in vitro* test, IL-6 in the supernatants of PBMCs stimulated with the "suspected" drugs with concentrations comparable on molar basis. The heterogeneity of the definition of positive drug allergy (positive response in drug patch test, or intradermal test, or drug provocation test) could be a possible weakness of the study.

## Endnotes

^a^In addition to Enalapril the following drugs gave only negative results: Acetylcystein, Ambroxol, Betaferon, Budesonid (2; epicutaneous test pos. in one) Chloropyramin, Drotaverin (3;1 false pos. in a control person), Famotidin, Ioversol, Clarythromycin, Pentasa, Salbutamol, Sulfametoxasol (3), Triamcinolon, Tramadol, Urapidin.

Automated serum IL-6 testing systems failed to detect PBMC released IL-6 although the standards for ELISA were detected with excellent linearity. The results of *Test A* medium were not influenced by lowering glucose concentration to 7 mM.

## Abbreviations

ACE: Angiotensin converting enzyme
ADR: Adverse drug reaction
AMX: Amoxicilline
BSA: Bovine serum albumin
CBA: Cytometric bead array
Con A: Concanavalin A
DMSO: Dimethyl-sulfoxyde
DRESS: Drug reaction with eosinophilia and systemic symptoms
EAACI: European Academy of Allergy and Clinical Immunology
EDTA: Ethylene-diamine-tetraacetic acid
ENDA: European Network for Drug Allergy
FACS: Fluorescence activated cell sorter
gp: Glycoprotein
IFN: Interferon
JAK/STAT: Janus kinase/signal transducer and activator of transcription
LDH: Lactate dehydrogenase
LTT: Lymphocyte Transformation test
MAPK: Mitogen-activated protein kinase
MEM: Minimal essential medium
MHC: Mean histocompatibility complex
O.D.: Optical density
PBMC: Peripheral blood mononuclear cell
PBS: Phosphate buffered saline
TCR: T cell receptor.
